# New Eco-Materials Derived from Waste for Emerging Pollutants Adsorption: The Case of Diclofenac

**DOI:** 10.3390/ma13183964

**Published:** 2020-09-07

**Authors:** Ario Fahimi, Alessandra Zanoletti, Stefania Federici, Ahmad Assi, Fabjola Bilo, Laura Eleonora Depero, Elza Bontempi

**Affiliations:** INSTM and Chemistry for Technologies Laboratory, Department of Mechanical and Industrial Engineering, University of Brescia, via Branze, 38, 25123 Brescia, Italy; a.fahimi@unibs.it (A.F.); alessandra.zanoletti@unibs.it (A.Z.); stefania.federici@unibs.it (S.F.); a.assi@unibs.it (A.A.); fabjola.bilo@unibs.it (F.B.); laura.depero@unibs.it (L.E.D.)

**Keywords:** emerging pollutants, diclofenac, adsorption, waste valorization, sustainability, eco-material, fly ash

## Abstract

This work proposes new eco-materials for the adsorption of diclofenac (DCF). The large consumption of this nonsteroidal anti-inflammatory drug combined with the inefficiency of wastewater treatment plants (WWTPs) leads to its presence in aquatic environments as an emerging pollutant. The adsorption technique is widely used for pharmaceutical removal. Moreover, due to the large effect of commercial adsorbents, in the frame of the Azure Chemistry approach, new sustainable materials are mandatory for removal as emerging pollutants. The work proposes three adsorbents that were obtained from different stabilization methods of fly ash derived from an incinerator plant; the stabilization techniques involved the use of various industrial by-products such as bottom ash, flue gas desulphurization residues, coal fly ash, and silica fume. The best performance, although less than activated carbon, was obtained by COSMOS (COlloidal Silica Medium to Obtain Safe inert: the case of incinerator fly ash), with a removal efficacy of approximately 76% with 15 g/L of material. Several advantages are expected not only from the DCF removal but also from an economic perspective (the newly obtained adsorbents are eco-materials, so they are cheaper in comparison to conventional adsorbents) and in terms of sustainability (no toxic reagents and no heating treatment are involved). This work highlights the adsorption performance of the new eco-materials and their potential use in WWTPs.

## 1. Introduction

The pharmaceutical market is constantly expanding, and Mezzelani et al. [1] estimated that the global production of pharmaceuticals will reach 4500 billion doses by the end of 2020. However, in the last several years, pharmaceutical have often been found in municipal wastewater [2]. As a consequence, scientists have categorized these contaminants as “emerging pollutants”. Indeed, they are present at low concentrations (from ng/L to µg/L) in wastewaters, ground waters, and surface waters (e.g., rivers or lacks). Moreover, although some scientists have been demonstrated the adverse effects on human health and environment of these pollutants, there is a lack of regulation [3]. Mostly, the effects regard the concentration, bioaccumulation, biotransformation, degradation, and persistence of the compounds. For example, the steroid hormone found in contraceptive pills persists in aquatic environments, even if it is present at low concentrations, preventing fishes from reproducing [4].

Organic pollutants are treated in conventional wastewater treatment plants (WWTPs) with physical (e.g., sedimentation, coagulation, and flocculation) and biological (e.g., activated sludge) processes. However, these processes may be ineffective in removing pharmaceuticals, because they occur at low concentrations with several chemical compositions [5]. Since it is necessary to improve the existing WWTP units, several studies report that the application of tertiary treatments (e.g., nanofiltration, ozonization, inverse osmosis, adsorption, and advanced oxidation processes (AOPs)) increase the removal efficiency of organic pollutants [2,6]. Although these additional processes would enhance the removal of the emerging pollutants, they may deal with some disadvantages: the high cost, the energy requirement [6], and the possible formation of more dangerous metabolites (e.g., the ones generated by AOPs) compared with the initial contaminants in WWTPs [7].

We focused our study on one emerging organic pollutant of interest in WWTPs, diclofenac (DCF), which is a nonsteroidal anti-inflammatory drug (NSAD) used to treat inflammatory and painful states in medical care [8]. It was recently added to the watch list under the Water Framework Directive (WFD). The literature estimates that the global annual consumption of DCF reaches 940 tons [9], and we expel approximately 65% of the DCF oral dosage via urine with its metabolites reaching the WWTPs through the wastewater [9]. Diclofenac is also very diffuse in surface water and groundwater, since its high stability in water and hydrophilicity make it able to persist in WWTPs [10,11]. The WFD monitors a list of priority substances to protect human health and environment for which the European Commission introduced the Environmental Quality Standards (EQS). The EQS derived the chronic toxicity data for the annual average (AA-EQS) and the acute toxicity data for the maximum allowable concentrations (MAC-EQS): the AA-EQS set the DCF limits to 0.1 μg/L and 0.01 μg/L for inland and coastal surface waters, respectively [12]; while, the MAC-EQS is set to 75 μg/L and 7.5 μg/L for inland and coastal surface waters, respectively.

**Figure 1:**
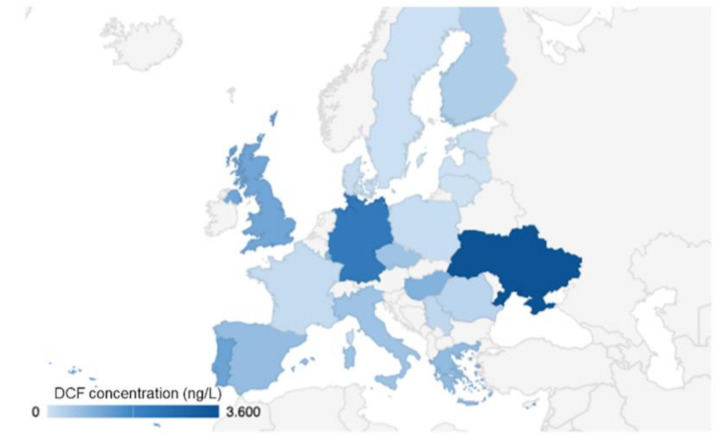
The occurrence of diclofenac (DCF) in European surface water. Data are taken from Reference [11] except for Denmark, Estonia, Latvia, Lithuania, and Poland where data are taken from Reference [13].

The literature reports different studies on DCF concentrations in surface water, groundwater, and sea water. Surface water is the category which needs to be steadily monitored, since it carries contaminants from several points: WWTP effluents, agricultural runoff, household waters, hospitals, and industrial sites. Sathishkumar et al. [11] reviewed the occurrence of DCF in different environmental compartments worldwide, and we used some data (as mean values of different locations for each country) for the European scenario to show the distribution of DCF concentrations in surface water as reported in Figure 1.

Some studies also exposed the toxicity of DCF for aquatic environments, plants, and mammals [11]. This compound can interfere with the biochemical function of fishes and also damage their organs (i.e., kidney or intestine) [4]. Indeed, some of the side effects are consistent with those caused by DCF in humans [14] and are demonstrated on mammals such as cardiotoxic neurotoxic or hepatoxic damages [11].

This study aimed to apply new adsorbents to remove DCF [4,15]. Adsorption on activated carbon is the most used technology to remove organic pollutants from different aqueous environments, but industries invest less and less due to the high cost and articulated regeneration process [16]. Therefore, novel alternative materials play a key role in order to compete with activated carbon in terms of adsorption capacity and rate, cost, and sustainability. The aim of this paper was to propose the use of new eco-materials, synthesized following the Azure Chemistry approach [17].

This work proposes new adsorbents derived from the stabilization of municipal solid waste incineration (MSWI) fly ash [18,19]. The stabilization method involved the use of different industrial by-products, resulting in a two-fold advantage: we recovered waste materials destined to landfill, and could exploit them as adsorbent materials—thanks to the stabilization reactions which enhanced the formation of a porous material—for improving the quality of water environments.

We analyzed experimental data for the adsorption mechanism describing the kinetics of the best adsorbent with different dosages (10 g/L, 15 g/L, 20 g/L, 50 g/L) under different models, i.e., pseudo first order, pseudo second order, and Elovich. In addition, as well as kinetics, we evaluated the adsorption equilibrium under different isotherm models, i.e., Langmuir, Freundlich, and Temkin. We also give some consideration to the environmental sustainability of a specific material, evaluating two parameters: the embodied energy (EE), that is the energy required to make 1 kg of the material from its ores or feedstocks, and the CO_2_ footprint (CF), that is the equivalent mass of greenhouse gases (kg CO_2_ equivalent) produced and released into the atmosphere after we produce 1 kg of material [20,21]. In this scenario, we could use data related to these parameters using the Cambridge Engineering Selector (CES) [22,23] to evaluate if novel materials derived from waste resulted in more environmentally sustainable material than commercial ones (i.e., activated carbon).

To our knowledge, no previous studies have applied stabilized MSWI fly ash for adsorption of DCF.

## 2. Materials and Methods

### 2.1. Materials

The incineration residues and industrial by-products involved in the preparation of adsorbents were fly ash (FA), bottom ash (BA), flue gas desulphurization (FGD) residues, coal fly ash (CFA), and silica fume (SF). Fly ash and BA were derived from a MSWI plant from A2A (Brescia, Italy) and represented the fine and coarse residues of MSWI, respectively. The FGD residues and CFA were provided by a coal thermal power plant located in the north of Italy. The SF consisted of by-products of silicon and ferro-silicon metal alloy processing and was provided by Metalleghe SPA (Brescia, Italy).

Powdered activated carbon (PAC, Darco, KB-G, CAS number: 7440-44-0) was purchased from Sigma–Aldrich (St. Louis, MO, USA).

### 2.2. Adsorbents Preparation

The adsorbents used were obtained by different stabilization methods of FA derived from an incinerator plant. The first method involved the use of SF, as an amorphous silica source, for the reduction of leachable heavy metals in the FA [18]. This method is a well-established technique, and the resulting inert material called COSMOS—COlloidal Silica Medium to Obtain Safe inert: the case of incinerator fly ash”—was patented in 2012 (patent number WO 2014020567 A1; [24]). The second method was recently developed in the frame of Rendering project “Recupero ENergetico dei fanghi di DEpurazione e loro Riutilizzo, IN alternativa ad alcune risorse naturali, per la produzione di compositi Green”. It provides the use of BA instead of SF as the main amorphous silica source [19].

Sample A and sample B were obtained by mixing under the following relative weight percentage (wt.%): on the one hand, sample A was characterized by 50% Milli-Q water, 30% FA, 9% FGD, 7% CFA, and 4% SF [25], and on the other hand, sample B was characterized by 48% Milli-Q water, 31% FA, 9% FGD, 7% CFA, and 5% BA [19]. As a comparison, the last sample (blank sample) was synthesized only by mixing in terms of wt.%: 50% Milli-Q water, 32% FA, 10% FGD, and 8% CFA. After 20 min of mixing powders with Milli-Q water, the samples were left at room temperature for a stabilization time equal to 2 months.

Leaching tests on these samples were performed in previous works, and the results confirmed the stabilization of the heavy metals initially contained in FA [19,26,27]. Before adsorption experiments, the materials were grinded with mortar, rinsed with Milli-Q water, filtered through a 0.45 µm filter, and finally dried at room temperature (T = 25 °C). The sample washing allowed to remove the presence of soluble salts on the surface of the material that could affect the performance of the adsorbent materials.

### 2.3. Characterization of Adsorbents

Structural characterization of adsorbents was performed by X-ray diffraction (XRD). The XRD measurements were performed with a X’Pert Pro diffractometer (PANalytical, Malvern, UK) equipped with a X’Celerator detector and Cu anode (CuKalpha 1.5406 Å) operating at 40 KV and 40 mA. The patterns were collected between 5° and 70° (in 2θ). To identify the phase composition, the PANalytical X’Pert HighScore Plus version 2.1 (associated with the International Centre for Diffraction Data (ICDD) Powder Diffraction File (PDF2) database, 1998) was used.

### 2.4. Adsorption Experiments

The DCF solution was prepared from Voltaren 75 mg/3 mL (Novartis, Basilea, Switzerland). Milli-Q water (Merck Millipore DirectQ-5 purification system, Molsheim, France) was used for the samples’ preparation and DCF solutions.

Five solutions with concentrations of 2.5, 5, 10, 15, and 28 mg/L of DCF were prepared and analyzed by QE65000 UV-Vis spectrophotometer (Ocean Optics) in order to determine the DCF calibration curve. Then, the concentration of DCF in the solution was evaluated by the UV-Vis analysis. The instrumental error was calculated performing 12 independent repetitions on a DCF solution (28 mg/L) and it was equal to 0.6%.

To evaluate the adsorption performance of the new adsorbents, for each material, four solutions of 20 mL of DCF with an initial concentration of (28.3 ± 0.2) mg/L were prepared and stirred for 6 h with 0.2 g, 0.3 g, 0.4 g, and 1 g of adsorbent, respectively. At different intervals of time—hour by hour —1.5 mL of the solution was removed and centrifuged at 11,000 rpm for 4 min in order to separate the adsorbent powder (which can interfere with the measurements) from the DCF solution and analyzed by UV-Vis spectrophotometer. The removal of DCF was calculated by Equation (1):

R (%) = (1 − C_t_/C_0_) × 100 (1)

where R is the percentage of DCF removal (%), C_0_ is the initial concentration of DCF (mg/L), and C_t_ is the residual concentration of DCF at time t (mg/L).

Based on the results, the adsorbent that showed the best performance for DCF removal was selected, and the equilibrium concentration C_e_ (mg/L) of each solution was evaluated. The amount of DCF adsorbed onto the material at equilibrium time q_e_ (mg/g) was calculated by Equation (2):

q_e_ = (C_0_ − C_e_) × (V/m) (2)

where C_0_ is initial concentration of the DCF solution (mg/L), C_e_ is the concentration at equilibrium of the DCF solution (mg/L), m is the mass of adsorbent (g), and V is the volume of the solution (L).

Finally, a comparison with activated carbon was tested; two DCF solutions of (28.9 ± 0.2) mg/L were prepared and mixed with 15 g/L of activated carbon and the selected material, respectively. The experiments were performed under the same conditions aforementioned for the three adsorbent materials.

## 3. Results and Discussion

### 3.1. Adsorbent Characterization

**Figure 2:**
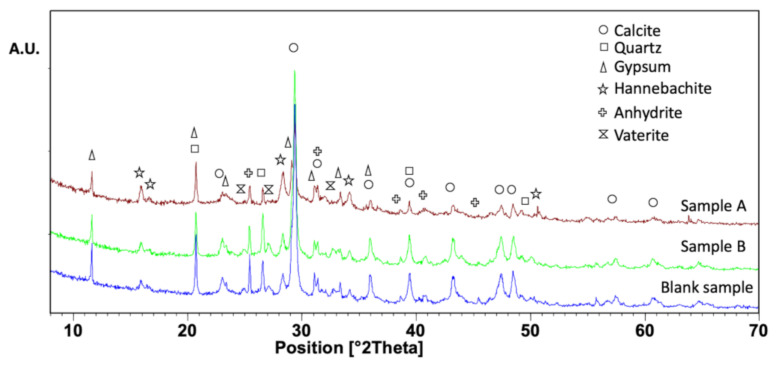
The XRD spectra of adsorbent materials: sample A, sample B, and a blank sample.

Figure 2 shows the XRD spectra of the three adsorbents. The identified crystalline phases are the following: calcite (CaCO_3_), quartz (SiO_2_), gypsum (CaSO_4*_ × H_2_O), hannebachite (CaSO_3_ × 0.5H_2_O), anhydrite (CaSO_4_), and vaterite (CaCO_3_). As reported in Reference [28], the presence of calcite (the main peak lies at 29.5°) and vaterite (a different polymorphic form of calcium carbonate) are attributed to carbonation reactions. As already reported [28], these materials also contain an amorphous phase. Gypsum, hannebachite, and anhydrite (the latter was identified mostly in the FA) represent different minerals of calcium sulfate with different hydration states and are all related to the use of FGD residues in the stabilization procedure.

### 3.2. Performance of Adsorbents

The three obtained eco-materials were tested against a solution containing DCF as a pollutant. We initially diluted a Voltaren solution from 75 mg/3 mL to about (28.3 ± 0.2) mg/L in order to make it comparable with the concentrations detected in several WWTPs (as seen in Figure 1). We diluted it at a range of concentrations measurable with the UV-Vis spectrophotometer in order to elaborate the calibration curve satisfying the Lambert–Beer law.

The calibration curve of DCF permitted to calculate directly the initial C_0_ of the stock blank solution, given the absorbance detected by the instrument before performing the adsorption tests. The calibration curve fits a straight-line equation that is reported in Equation (3). The linear regression coefficient of determination (R^2^) was approximately 0.99679.

y = 0.0352 x − 0.0195(3)

where y is the DCF absorbance, and x is the DCF concentration.

**Figure 3:**
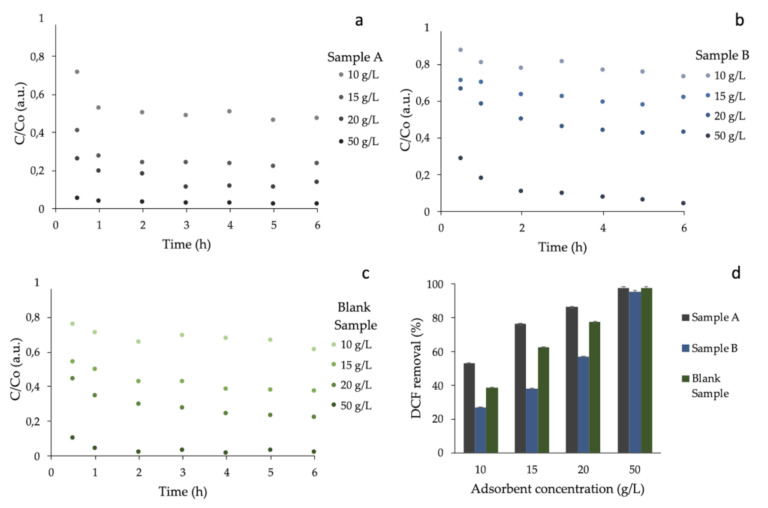
Variation of DCF concentrations (normalized to C_0_) with respect to the variation of time at 4 different adsorbent amounts for sample A (**a**), sample B (**b**), and a blank sample (**c**). We also reported the comparison of DCF removal (%) at the end of each experiment (t = 6 h) for the three materials (**d**). An instrumental error equal to 0.6% for the experimental data was considered. Error bars are included within the experimental point size (a, b, and c).

Figure 3a–c shows the variation of DCF concentrations at different times in the presence of sample A, sample B, and a blank sample, respectively. Figure 3d reports the comparison of DCF removal for the three materials after 6 h of adsorption time. As reasonable, the increase of adsorbent concentration generated an increase of the DCF adsorption. We can observe for all cases how increasing the dose of adsorbent led to an increasing uptake of the pollutant. Approximately 97% of DCF removal was reached with 50 g/L for all adsorbents. The best performance was obtained by the COSMOS material (sample A). In fact, at low concentration of adsorbent, a higher adsorption removal was obtained. We noticed that sample A was the only case in which all the adsorbents’ concentrations removed at least 50% of DCF within 1 h. In another example, considering 15 g/L of COSMOS, DCF was removed from water efficiently at 76% (see Figure 3a). Moreover, we highlight that the adsorption took place quickly: two out three materials reached the maximum adsorption and then stabilized after approximately two hours (we strictly assumed that the equilibrium time was achieved within 6 h).

We must remind that the realization of this sample took place with different by-products. Firstly, the literature reports that CFA is rich in unburned carbon that is responsible for the adsorption of a large category of pollutants [29]; in fact, the performance of the blank sample showed how CFA is capable of adsorbing DCF by itself, and this is also confirmed in Reference [30] where CFA was tested to adsorb sodium dodecyl sulphate which is a model of anionic surfactants. Secondly, SF is also involved in the adsorption of DCF; the silica surface is characterized by the presence of silanol groups (Si–OH) that trigger adsorption sites due to the presence of mechanisms such as anion exchange and hydrophobic partitioning of DCF [31,32,33].

Kinetic studies were performed by selecting sample A, due to the fact of its better efficiency relatively to the other two materials (see Figure 3d).

### 3.3. Kinetics

**Figure 4:**
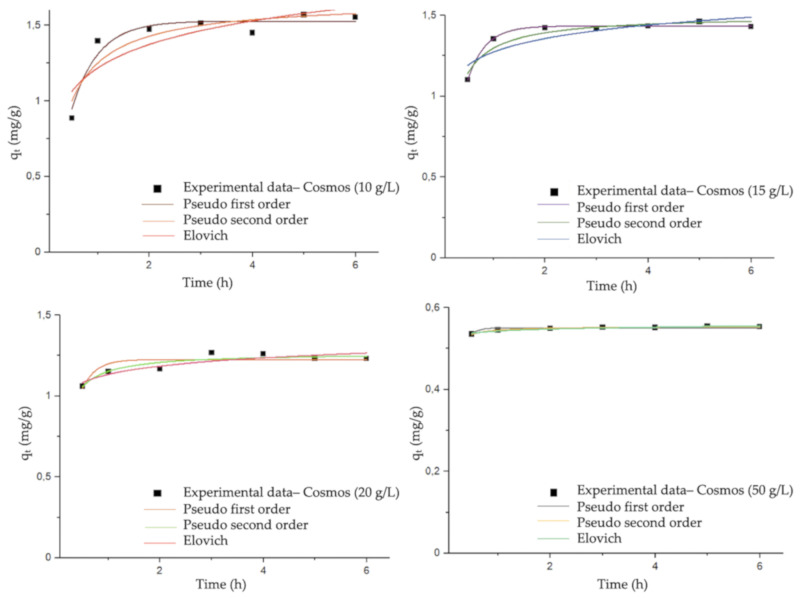
Nonlinear kinetic models for the adsorption of DCF onto COSMOS (sample A) at different dosages (10 g/L, 15 g/L, 20 g/L, 50 g/L). The fit was performed by Origin v.15.0 data analysis software (OriginLab Corporation, 2020), employing a Levemberg–Marquardt iteration algorithm.

**Figure 5:**
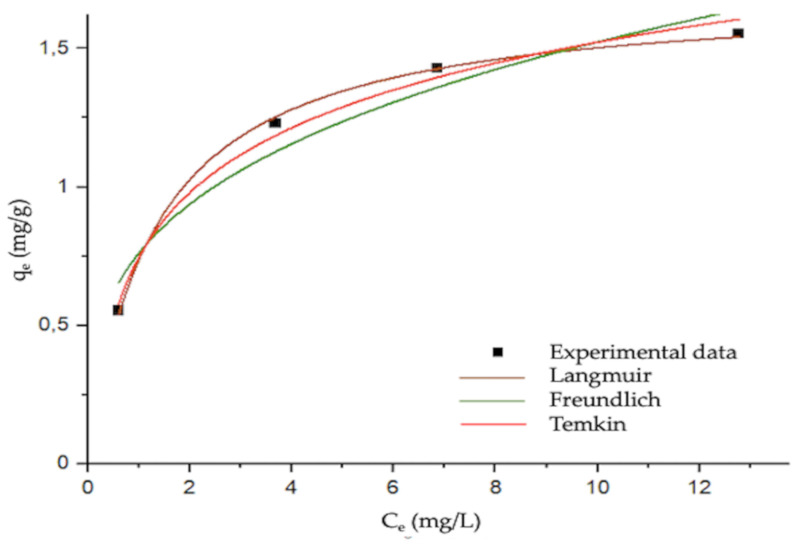
Nonlinear isotherms for the adsorption of DCF onto COSMOS (sample A). The isotherm was reported at a fixed T (room temperature equal to 298 K). The fit was performed by Origin v.15.0 data analysis software (OriginLab Corporation, 2020), employing a Levemberg–Marquardt iteration algorithm. Fitting equations and value parameters are reported in Table 2.

The kinetic study evaluated the interaction of DCF and the sample A, and it investigated the mechanism of chemical sorption via calculating the most suitable rate constant (among different models proposed in literature) to fit experimental data. The mechanism of adsorption was analyzed with three different models: pseudo first order, pseudo second order, and the Elovich model as visible in Figure 4. The parameters for each model, according to References [34,35,36] and the coefficients R^2^ (indicative value for the fitting) are represented in Table 1. Based on the coefficient R^2^, it is clearly shown that the adsorption of DCF follows the pseudo first order model for the COSMOS (sample A) as long as the dosage of material stays within 15 g/L. With the increasing quantity of adsorbent material, the adsorption changed its kinetic mechanism to a pseudo second order model. Both models were characterized by the parameters q_1_ (mg/g) and q_2_ (mg/g), which are the amounts of DCF adsorbed onto COSMOS at an equilibrium time for pseudo first order and pseudo second order, respectively, and q_t_ (mg/g), which is the amount of DCF adsorbed onto COSMOS at time t. In the same way, K_1_ (1/h) and K_2_ (g/mg × min) are the equilibrium rate constant of the pseudo first order model and pseudo second order model, respectively. It is fundamental that the best fitted model for each dosage shows a good agreement with the value of q_e_ experimentally obtained at equilibrium; in the present work, the values were in line with the experimental data shown in the isothermal plot in Figure 5.

### 3.4. Equilibrium Isotherm

The evaluation of isotherm has a strategic meaning in view of developing an equation that could be exploited for design purposes.

The equilibrium concentrations C_e_ (mg/L) were identified considering the residual concentration of DCF at t = 6 h after checking the evolution of C_t_ with the variation of t (see Figure 3). Then, from the mass balance equation at equilibrium, which derived from the operating line predicting the t dependence of the adsorption mechanism and integrating to an equilibrium state, we can substitute C_e_ in the Equation (2). Then, from the calculated q_e_ coupled with C_e_ we could obtain the isotherm in Figure 5 that shows how the model is associable with the Langmuir model. The Langmuir model assumes that every adsorption is homogeneous, it occurs in monolayers, and every active site has equivalent energy [37]. It is worth highlighting how the q_e_ values (see ordinate values reported in Figure 5) are close to the q_2_ (see Table 1) obtained for the kinetic model, and this validates the choice of the equilibrium t = 6 h.

At a low value of C_e_, there is a rapid increase of q_e_ until the plateau is reached. This plateau regards high concentrations indicating the saturation of the adsorbent. The saturation can be calculated from the nonlinear fitting approach evaluating principally three models: Langmuir, Freundlich, and Temkin. The adsorption parameters, the coefficients of determination *R*^2^, and the respective equations are reported in Table 2. Langmuir had the highest coefficient of determination (*R*^2^ = 0.9986) better describing the equilibrium process than Freundlich (multilayer adsorption on a heterogeneous surface). Langmuir *R*^2^ was not far from the *R*^2^ related to the Temkin model which was equal to 0.9889, and this model described the interaction between the adsorbate and the adsorbent as a cause of the linear decrease in the adsorption heat for the molecules onto the layer during the adsorption process.

Taking into account the best fit, K_L_(L/g) and q_max_ (L/mg) are the Langmuir constant and the maximum adsorption capacity (the saturation of the adsorbent), respectively; thus, the maximum DCF adsorbed onto the COSMOS q_max_ was 1.7 mg/g (see Table 2). The K_L_ is thermodynamically associable with the Gibbs free energy in an equilibrium process, and it can be used in order to evaluate if the adsorption process occurs spontaneously. Based on the Van’t Hoff’s isothermal equation, we obtained Equation (4):

∆G = ∆G° + R × T × ln (K_L_) (4)

where, per definition, ΔG is the Gibbs free energy change of the overall adsorption process (we are discussing the equilibrium state; thus, it means ∆G = 0), ∆G° is the Gibbs free energy change for unmixed solute, the adsorbent at standard conditions (T = 298 K, p = 100 kPa), R is the universal gas constant (8.314 J/mol K), T is the temperature under which the adsorption process undergoes (in our case it is equal to 298 K), and finally K_L_ is the thermodynamic equilibrium constant (K_L_ = 0.76 L/g) that represents the current condition relative to the state of a system. Rearranging the equation, we obtain Equation (5):

∆G° = − R × T × ln (K_L_) = − 1.88 kJ/mol (5)

From the negative value of ∆G°, we can possibly state that the adsorption process occurs spontaneously.

### 3.5. Comparison with Activated Carbon

**Figure 6:**
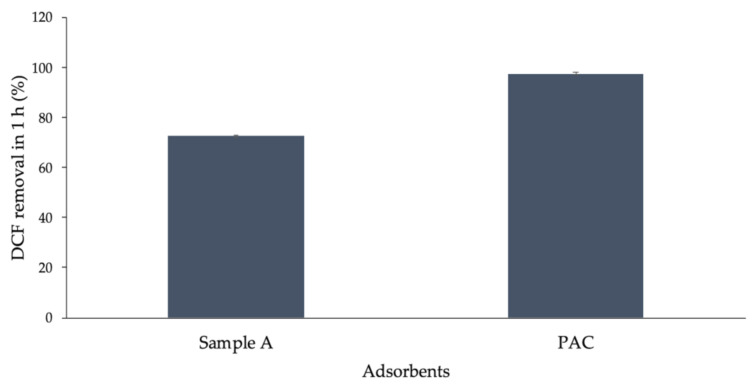
Diclofenac removal (R%) for sample A compared with powdered activated carbon (PAC) in 1 h test. An instrumental error equal to 0.6% for the experimental data was considered.

Following the results in Section 3.2, sample A was selected for the comparison with activated carbon. Two DCF solutions of (28.9 ± 0.2) mg/L were, respectively, mixed with 15 g/L of activated carbon and sample A. Results showed (see Figure 6) that the DCF removal (calculated with Equation (1)) reached 98% with activated carbon while 72% with sample A within 1 h test. This is a promising outcome in terms of performance; if we optimize the crushing method, we may obtain a material with a higher surface area which positively influences the initial adsorption rate as Huggins reports [38]. Furthermore, we do not have to deal with end-life issues as for activated carbon. On the one hand, activated carbon faces the regeneration process at the end of its life-cycle in order to be able to do its task again; on the other hand, COSMOS could be used directly for other applications (e.g., as filler in cement production) at the end of its life-cycle, resulting in some capital and energy, even in end-life use compared with activated carbon.

Therefore, COSMOS is a possible candidate as a substitute of activated carbon for engineering applications, even though it is difficult to predict the economic effects because of many factors, e.g., the period of time under analysis, market dimensions, energy supply of a country, and so on.

On the contrary, the environmental sustainability of sample A can be evaluated in terms of embodied energy (EE) and carbon footprint (CF) by a new simplified approach [21]; EE and CF values were evaluated using data provided by the CES Selector and reported in previous works [21,30]. Based on this approach, we classified COSMOS as an eco-material: it requires only by-products, a negligible amount of energy for production, and finally a sustainable stabilization procedure (i.e., no toxic reagents and thermal treatments). Therefore, we estimated that COSMOS has a lower EE and CF (0.82 MJ/Kg and 0.02 kg CO_2−eq_/kg, respectively) in comparison to activated carbon (43.4–277 MJ/kg and 3.96–22.0 kg CO_2−eq_/kg) [39], indicating that COSMOS results in a more promising and convenient material than activated carbon.

## 4. Conclusions

This work reports on the evaluation of the adsorption of DCF, as a model for emerging micropollutants, by using new eco-materials generated from different stabilization methods starting with MSWI fly ash. Adsorption experiments revealed good performance for these materials, although less than activated carbon. The best performance was obtained by COSMOS, with a removal efficacy of approximately 76% with 15 g/L of material. The advantages are expected not only from environmental point of view (the DCF removal from water) but also from an economic and sustainable point of view. The analysis of EE and CF of the proposed eco-materials showed that they are the most sustainable in comparison with activated carbon. The new adsorbent material can be applied in WWTPs, offering new prospects in the field of micropollutants removal.

## Figures and Tables

**Table 1:** Nonlinear kinetic parameters for the adsorption of DCF onto COSMOS (sample A) for all the dosages (10 g/L, 15 g/L, 20 g/L, 50 g/L).

Isotherm Model	Equation	Parameter	Value			
			10 g/L	15 g/L	20 g/L	50 g/L
Pseudo first order	q_t_ = q_1_ × (1 − e ^(− K^_1_^× t)^)	q_1_ (mg/g)	1.53 ± 0.03	1.43 ± 0.01	1.23 ± 0.02	0.551 ± 0.001
		K_1_ (1/h)	1.94 ± 0.2	2.91 ± 0.09	3.85 ± 0.5	7.06 ± 0.44
		R^2^	0.93915	0.98931	0.73305	0.80547
Pseudo second order	q_t_ = K_2_ × (q_2_^2^) × t/(1 + (K_2_ × q_2_ × t))	q_2_ (mg/g)	1.66 ± 0.07	1.5 ± 0.02	1.27 ± 0.02	0.56
		K_2_ (g/(mg × h))	1.82 ± 0.54	4.21 ± 0.72	7.9 ± 1.6	96.7 ± 6.9
		R^2^	0.86669	0.94351	0.87274	0.97751
Elovich	q_t_ = (1/β) × ln (1 + (α × β × t))	α (mg/(g × h))	50.85 ± 73.2	4950 ± 13066	365940 ± 1206882	1.34 × 10^31^ ± 1.34× 10^32^
		Β (g/mg)	4.5 ± 1.2	8.35 ± 2.1	13.6 ± 2.9	141 ± 19
		R^2^	0.74413	0.76106	0.81197	0.92076

**Table 2:** Nonlinear isotherm models for the adsorption of DFC onto Cosmos (sample A).

Isotherm Model	Equation	Parameter	Value
Langmuir	q_e_ = (q_max_ × K_L_ × C_e_)/(1 + (C_e_ × K_L_))	q_max_ (mg/g)	1.7 ± 0.02
		K_L_ (L/mg)	0.76 ± 0.05
		R^2^	0.99864
Freundlich	q_e_ = K_F_ × (C_e_^(1/n)^)	K_F_ ((mg/g) (L/mg)^1/n^)	0.76 ± 0.1
		n	3.32 ± 0.71
		R^2^	0.94351
Temkin	q_e_ = (R × T/b) × ln (K_T_ × C_e_)	K_T_ (L/mg)	9.09 ± 2.53
		B (kJ/mol)	7334 ± 552
		R^2^	0.9889
